# 1115. Case Study. Case Report of a 7-year-old Boy with Severe Erythema Multiforme Major

**DOI:** 10.1093/jbcr/irag033.289

**Published:** 2026-04-06

**Authors:** Zachary Brown, Michael J Feldman

**Affiliations:** Evans-Haynes Burn Center at VCU Health, Richmond, VA; Virginia Commonwealth University, Richmond, VA

## Abstract

**Patient Presentation (age range, injury details, relevant history):**

A 7 year old boy presents with fever and cough worsening over four days, and crusting of the lips and scant erythematous lesions to his right lateral chest wall on day five, becoming raised and pruritic the next day. His pediatrician diagnosed mycoplasma pneumonia and prescribed azithromycin. Despite two doses, he developed an increased number of cutaneous lesions and blistering involving bilateral eyelids and conjunctiva prompting presentation to the emergency room. There he had periorbital edema and erythema with eyelid margin crusting; hemorrhagic crusting over his lips; and scattered targetoid papules and plaques, some with central dusky discoloration and vesiculation, to the face, torso, bilateral upper and lower extremities with sparing of the palms and soles, and genitalia, covering 30% total body surface area. These lesions eventually became bullae and sloughed. On hospital day 16 he experienced intermittent periumbilical pain and imaging demonstrated right colon pneumatosis intestinalis.

**Clinical Challenges:**

There are many overlapping etiologies, symptoms, and clinical features of reactive infectious mucocutaneous eruption and erythema multiforme major with the main differentiation seeming to be severity. They are indistinguishable histologically.

**Management Approach:**

Admission to pediatric intensive care for pain control. Burn surgery, ophthalmology, and dermatology were consulted. Cutaneous lesions developed prior to antibiotics, so Stevens-Johnson syndrome/toxic epidermal necrolysis was considered less likely. Labwork was significant for positive *Mycoplasma pneumoniae* IgM and IgG, though indirect fluorescent antibodies were not present; respiratory PCR panel was negative for all tested pathogens; and serology was negative for Epstein–Barr, HSV-1 and 2, and HHV-6. Skin biopsy was not performed as the two likely pathologies are indistinguishable histologically. Patient was treated systemically with azithromycin, acyclovir, and cyclosporine. Daily wound care performed, with sedation as indicated. His ocular lesions were treated with neomycin/polymyxin B/dexamethasone drops; facial and genitalia lesions were treated with hydrocortisone; and all other areas were treated with mupirocin and triamcinolone. His pneumatosis intestinalis was managed with NPO status, cefepime, and metronidazole for five days.

**Outcomes:**

Patient discharged home on hospital day 25, eventual complete clinical resolution.

**Lessons Learned:**

The overlapping symptoms, treatment strategies, and indistinguishable histologies for the two diseases supports the hypothesis that they may represent a continuum of disease.

**Applicability to Practice:**

Erythema multiforme major affects 0.01-1% of the population. It is rare to see so severe a presentation, especially in a child. This case report will increase awareness and provide guidance on diagnosis and treatment.

